# Unfolded Protein Response Pathways Correlatively Modulate Endoplasmic Reticulum Stress Responses in Rat Retinal Müller Cells

**DOI:** 10.1155/2019/9028483

**Published:** 2019-02-24

**Authors:** Shengyu Wu, Xiaolu Zhu, Biechuan Guo, Tian Zheng, Jiangbo Ren, Wen Zeng, Xiaomin Chen, Min Ke

**Affiliations:** Department of Ophthalmology, Zhongnan Hospital of Wuhan University, Wuhan, Hubei 430071, China

## Abstract

**Background:**

Endoplasmic reticulum stress (ERS) in the retinal Müller cells is a key factor contributing to the retinal inflammation and vascular leakage in diabetic retinopathy (DR). This study was to investigate the underlying mechanisms through which the 3 main unfolded protein response (UPR) pathways regulate ERS and to examine the expression levels of vascular endothelial growth factor (VEGF) in Müller cells *in vitro*.

**Methods:**

Rat Müller cell lines were stimulated with high glucose to mimic a diabetic environment *in vitro*. PKR-like endoplasmic reticulum kinase (PERK), inositol-requiring enzyme 1 (IRE1) and activating transcription factor 6 (ATF6) were downregulated or upregulated with shRNA or overexpression plasmids. The transfected Müller cells were cultivated in high glucose medium for 48 hours. Expression of glucose-regulated protein 78 (GRP78), activating transcription factor 4 (ATF4), X-box binding protein 1 (XBP1), ATF6, and VEGF was examined with immunofluorescence and western blot.

**Results:**

Our data indicated that ERS was found in both high glucose and osmotic control groups. Overexpression or downregulation of UPR pathways effectively increased or reduced the production of GRP78, ATF4, XBP1, ATF6, and VEGF, respectively. These 3 signaling pathways had similar regulatory effects on VEGF.

**Conclusion:**

The 3 UPR-mediated inflammatory pathways were dependent on each other. Inhibition any of these signaling pathways in UPR might be a potential therapeutic target for DR.

## 1. Introduction

Diabetic retinopathy (DR) is a severe complication of diabetes and one of the leading causes of binocular blindness [1]. Its incidence increased considerably along with diabetes during last decades [2]. The global incidence of DR is predicted to rise dramatically from an estimated 127 million people in the year 2010 to 191 million by 2030 [3]. Several treatments are available for DR, including photocoagulation [4], vitrectomy [5], and repeated intraocular injections of steroids [6]; however, adverse effects are common [7].

Müller cells are principal glial cells in the retina to maintain retinal homeostasis [8]. They are rich in endoplasmic reticulum (ER) and express growth factors to nourish retinal neurons and capillary cells [9]. Vascular endothelial growth factor (VEGF) is produced by Müller cells [10] in the retina and activated at the early stages of DR [11]. Anti-VEGF therapy, as a major strategy to treat DR, was demonstrated to alleviate retinal inflammation and vascular leakage in diabetic patients [12–14]. However, inhibition of VEGF by anti-VEGF antibodies resulted in systemic adverse effects such as thromboembolic events, hemorrhage, gastrointestinal perforation, hypertension, and nephrotic syndrome [15]. Therefore, investigation of the key mechanisms that regulate inflammation in Müller cells may elucidate new therapeutic targets to prevent retinal complications of diabetes.

The ER is the primary cellular organelle responsible for the synthesis and processing of proteins [16]. Protein folding in the ER may be disturbed by various physiologic and pathologic conditions, causing endoplasmic reticulum stress (ERS) [17]. To restore homeostasis under ERS, cells usually trigger the unfolded protein response (UPR), which alleviates the accumulation of misfolded or unfolded protein [18]. UPR is transduced by 3 main ER-resident stress sensors, PKR-like endoplasmic reticulum kinase (PERK) [19], inositol-requiring enzyme (IRE) 1 [20], and activating transcription factor (ATF) 6 [21]. At normal condition, these 3 proteins are bonded to glucose-regulated protein (GRP) 78 associated with the internal membrane. Upon ER stress, GRP78 is upregulated rapidly and then dissociates from the ER membrane to enter the lumen [22]. Previous studies demonstrated that high glucose induced ERS in Müller cells [23]. VEGF, as an important molecule signaling preinflammation, was apparently induced by ERS [10].

Taken together, high glucose induced retinal ERS reactions. However, the underlying mechanisms and signaling pathways in UPR-mediated stress are yet to be determined. In this study, we used a hyperglycemia cell model to simulate the diabetic environment [24], explored the UPR pathways, and investigated the mechanisms that activated ERS in rat retina Müller cells.

## 2. Materials and Methods

### 2.1. Cell Culture

Rat retinal Müller cell line (rMC-1) was generously provided by the College of Life Sciences at Wuhan University (Wuhan, China). Cells were cultured in Dulbecco's modified Eagle's medium (DMEM) containing 1 g/l glucose with 10% fetal bovine serum, 1% penicillin, and 1% streptomycin, at 37°C in a humidified atmosphere with 5% CO_2_. After washing with 0.25% Tris in the logarithmic growth phase, cells were seeded in 24-well polystyrene plates. The cells were then verified by immunocytochemistry of glial fibrillary acidic protein (GFAP; DAKO, Hamburg, Germany). For the studies of high glucose (HG), cells were starved in serum-free DMEM overnight and treated with or without normal glucose (5 mmol/L), high glucose (30 mmol/L), or mannitol (25 mmol/L; as an osmotic control) for 48 hours.

### 2.2. Construction of shRNA Plasmid

Short hairpin RNAs (shRNAs) were designed according to the ATF6, PERK, and IRE1 mRNA sequences in the GenBank. Oligonucleotides were synthesized by GenePharma Co., Ltd (Shanghai, China). The annealed shRNA oligonucleotides were ligated into pYr1.1 vector between the XhoI and EcoRI sites by T4 DNA ligase (Fermentas, USA) according to the manufacturer's protocol. The ligated products were transformed into competent *E.coli* competent cells according to *Molecular Cloning: A Laboratory Manual (3rd edition)* [25]. The transformed bacteria were grown on a LB-agar plate containing 50 *μ*g/ml kanamycin. Screening of positive clones was done by colony PCR using specific primers:

  shRNA-ATF6-242 F-CCTTGGGAGTCAGACATAT, R-ATATGTCTGACTCCCAAGG;
  shRNA-ATF6-682 F-GCAGTCGATTATCAGTATA, R-TATACTGATAATCGACTGC;
  shRNA-ATF6-1601 F-GCTGTCCAGTACACAGAAA, R-TTTCTGTGTACTGGACAGC;
  shRNA-PERK-1216 F-GCTGGTGAGGGATGGTAAA, R-TTTACCATCCCTCACCAGC;
  shRNA-PERK-2001 F-GCGTTGTCTTTGAAGCTAA, R-TTAGCTTCAAAGACAACGC;
  shRNA-PERK-2859 F-GCAGGAAGGAGAACCTTAA, R-TTAAGGTTCTCCTTCCTGC;
  shRNA-IRE1-1173 F-GGAGGTTATCAACCTAGTT, R-AACTAGGTTGATAACCTCC;
  shRNA-IRE1-2219 F-CCTACACAGTGGACATCTT, R-AAGATGTCCACTGTGTAGG;
  shRNA-IRE1-2732 F-GCTCCATCCCTGATGACTT, R-AAGTCATCAGGGATGGAGC.

The positive clones were subcultured to isolate the plasmid DNA by Rapid Plasmid Max iPrep Kit (TIANGEN, USA). The validated shRNA plasmids were transferred into rMCs for the following experiments.

### 2.3. Construction of pEGFP-RNA Plasmid

Total RNA was extracted from rMCs using Trizol (Invitrogen, USA). The reverse transcription reaction was conducted using a reverse transcription kit (Thermo Fisher Scientific, USA) to obtain cDNA according to the manufacturer's protocols. The recombinant pEGFP-N1-ATF6 and pEGFP-N1-PERK plasmids were constructed at HindIII and XhoI restriction endonuclease sites and validated by Sangon Biotech (Shanghai). The pEGFP-IRE1 plasmid was purchased from Zoonbio Biotechnology (Nanjing, China).

### 2.4. Transfection

Rat Müller Cells were transiently transfected in duplicates in 6-well plates using Lipofectamine 2000 (Invitrogen), following the manufacturer's protocol. The cells (2 × 10^5^ per well) were incubated with fresh DMEM medium without antibiotics for 48 hours to obtain 80–90% confluency on the day of transfection. Mock-transfected cells (i.e., cells transfected with Lipofectamine reagent only) served as negative controls.

### 2.5. Immunofluorescence Assay

Rat Müller cells cultured on a 6-well chamber were washed twice with PBS and fixed with 4% paraformaldehyde in PBS for 10 min, followed by penetration with Triton X-100 (0.1%, Invitrogen) for 15 minutes. The cells were blocked with 5% BSA (Invitrogen) in PBS for 1 hour and incubated with primary antibodies at 4°C overnight. The cells were then incubated with secondary antibodies (1 : 200; Cy3-labled goat anti-rabbit IgG, Boster Biological Technology, Wuhan, China) at room temperature for 60 minutes. After washing with PBS three times (2 min/time) and staining with DAPI to visualize nuclei, the cells were analyzed under a fluorescence microscope (Olympus, Japan). Primary antibodies used rabbit anti-mouse glial fibrillary acidic protein (GFAP, Thermo Fisher Scientific), anti-XBP1s (1 : 600, Proteintech, USA), anti-ATF4 (1 : 800, Proteintech), and anti-ATF6 (1 : 200, Proteintech).

### 2.6. Quantitative Real-Time Polymerase Chain Reaction (qRT-PCR)

Total RNA was extracted from rMCs using Trizol. RNA (2 *µ*g) was used for cDNA synthesis using a Revert Aid First Strand cDNA Synthesis Kit (Invitrogen). Quantitative real-time PCR was performed with Fast Start Universal SYBR Green Master Kit (Rox, USA) on a QuantStudio 5 Real-Time PCR system (Applied Biosystems, USA). The experiments were independently repeated 3 times. PCR primer sequences were as follows:

  ATF6-F: 5′-ATGGAGTCGCCTTTTAGTCC-3′;
  ATF6-R: 5′-CTGTACCGACTCAGGGAGGG-3′;
  PERK-F: 5′-AAGGCTCCTAGCGGCGAGAC-3′;
  ATF6-R: 5′-CGTTGCCAGGCAGTGGGCTGA-3′;
  IRE1-F: 5′-GCGATGGACTGGTGGTACT-3′;
  IRE1-R: 5′-GTTTGCTCTTGGCCTCTGTC-3′.

The comparative *C*_T_ method was used to calculate the mRNA expression levels. The expression of selected genes was normalized to that of the reference gene, *β*-actin, at each time point and converted to the relative levels as follows: fold expression = 2ΔΔ*C*_T_, where Δ*C*_T_ = average *C*_T_ of target gene − average *C*_T_ of endogenous control (*β*-actin) and ΔΔ*C*_T_ = average Δ*C*_T_ of target sample − average Δ*C*_T_ of the calibrator sample.

### 2.7. Western Blot Analysis

Rat Müller cells were washed with PBS and lysed with RIPA lysis buffer (Beyotime, China) on ice for 30 minutes. The protein concentrations were detected by the BCA protein assay (Beyotime). The proteins were separated in 4–6% sodium dodecyl sulfate polyacrylamide gel electrophoresis (SDS-PAGE, Beyotime, China) and then transferred to a PVDF membrane (Beyotime). After blocking with 5% nonfat milk in TBST buffer (200 mM Tris and 1.5 M NaCl with 0.1% Tween 20), the membranes were blotted at 4°C overnight with the following primary antibodies: anti-XBP1s (1 : 600; Proteintech), anti-GRP78 (1 : 800; Proteintech), anti-VEGF (1 : 600; Proteintech); anti-ATF4 (1 : 800; Proteintech), and anti-ATF6 (1 : 200; Proteintech). The membranes were sequentially probed with horseradish peroxidase-conjugated goat anti-rabbit antibody (Boster Biological Technology) at room temperature for 2 hours. *β*-Actin was served as the loading control. Enhanced chemiluminescence (ECL, Beyotime) was used for imaging, and finally, the optical density of the band was analyzed by GeneTools software (Syngene, Synoptic Ltd. USA). All experiments were repeated for 3 times independently.

### 2.8. Statistical Analysis

All data were presented as mean ± standard deviation (SD). Comparisons between 2 groups were conducted with Student's *t*-test. The average number of samples was compared using single-factor analysis of variance (ANOVA). Multiple comparisons between the groups were tested by the SNK test. *P* < 0.05 was considered as statistically significant.

## 3. Results

### 3.1. Purity of Müller Cell Culture

**Figure 1:**
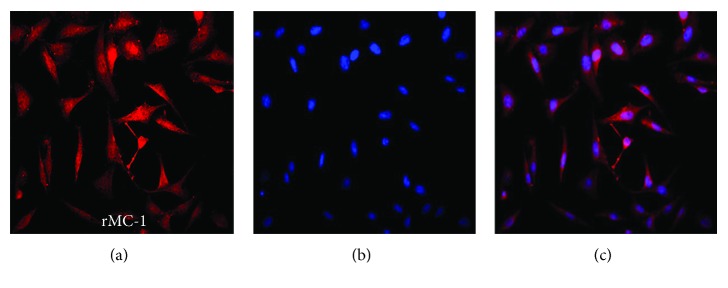
Rat Müller cells were stained with Müller cell-specific marker GFAP. (a) GFAP. (b) DAPI. (c) Merge.

Immunocytochemistry was used to determine the purity of Müller cell cultures. The intermediate filament protein GFAP was reported to express in astrocytes and Müller cells [26]. In our study, the cultured cells had strong GFAP staining (Figure 1). The results showed 95% cells were GFAP-positive and derived from Müller cells.

### 3.2. Detection of ERS Markers and VEGF in rMCs Exposed to HG for 48 h

**Figure 2:**
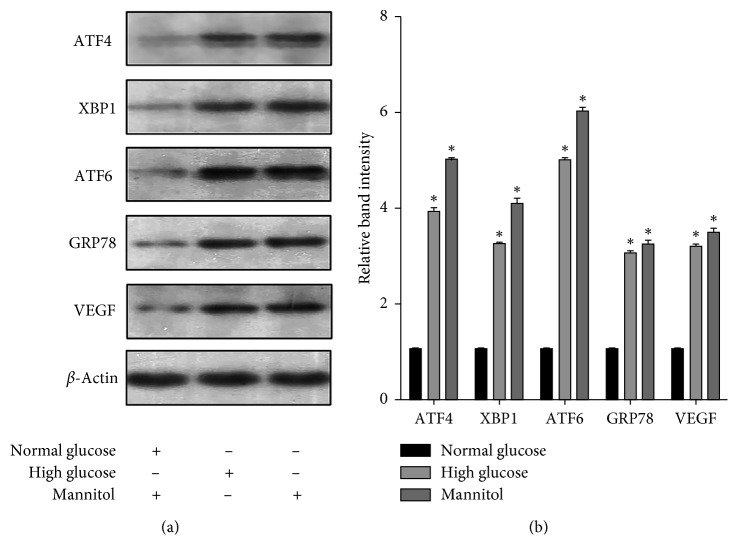
HG induced ER stress and increased expression of VEGF in rMCs. Expression of ER stress markers and VEGF in rMC-1 cells when exposed to normal glucose (5 mmol/L), HG (30 mmol/L), or glucose (5 mmol/L) plus mannitol (25 mmol/L) for 48 hours was determined by western blot analysis. The results were representative of 3 independent experiments (*n*=3; ^*∗*^*p* < 0.05).

Our preliminary studies demonstrated that ERS was not fully activated by HG at 24 hours, so we extended the intervention to 48 hours. At this time point, the protein levels of the major markers for ER stress were increased significantly compared with the rMCs exposed to normal glucose (*p* < 0.05, Figure 2). Moreover, VEGF was also markedly upregulated (*p* < 0.05), suggesting induced retinal neovascularization, vascular leakage, and perhaps macular edema in DR [27]. Interestingly, our results also indicated that rMCs grown in glucose (5 mmol/L) plus mannitol (25 mmol/L) showed a significant increase in the expression of ER stress markers and VEGF (*p* < 0.05).

### 3.3. Construction of shRNA and Overexpression Plasmid

**Figure 3:**
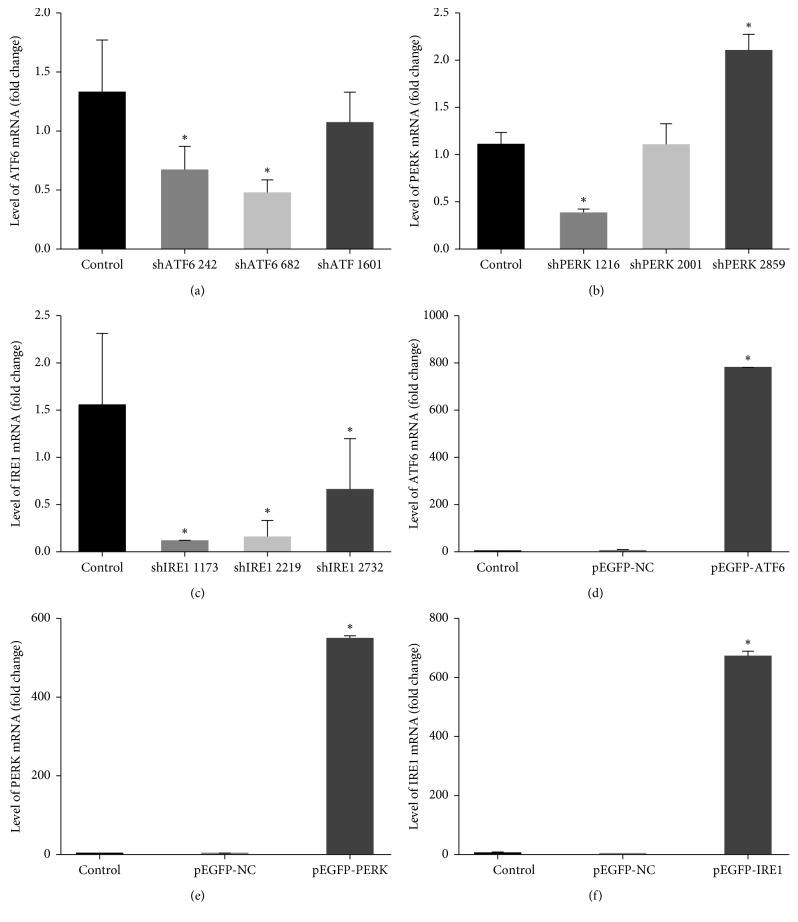
Transcriptional modulation by UPR pathway regulation in rMCs. The amounts of ATF6, IRE1, and PERK mRNAs in rMCs with shRNA and overexpression plasmids were determined by PCR analysis. (a) ATF6 silencing with 3 shRNAs (shATF6 242, shATF6 682, and shATF6 1601). (b) PERK silencing with 3 shRNAs (shPERK 1216, shPERK 2001, and shPERK2859). (c) IRE1 silencing with 3 shRNAs (shIRE1 1173, shIRE1 2219, and shIRE1 2732). (d) The mRNA levels of ATF6 after pEGFP-ATF6 transfection in rMCs. (e) The mRNA levels of PERK after pEGFP-PERK transfection in rMCs. (f) The mRNA levels of IRE1 after pEGFP-IRE1 transfection in rMCs. All experiments were performed in triplicates (control (mock transfected); ^*∗*^*p* < 0.05).

In order to investigate the ATF6 response, 3 different ATF6-shRNAs were designed and their effects were examined by quantitative real-time PCR. All of the 3 shATF6s downregulated ATF6 mRNA levels (Figure 3(a)) in transiently transfected rMC-1, compared with the control group. (*p* < 0.05). Moreover, shATF6 682 had the strongest downregulated effects. Similarly, 3 shPERKs were transfected into rMCs, and shPERK 1216 showed significant reduction (*p* < 0.05) on the PERK mRNA levels compared with the control group (Figure 3(b)). The shIRE1 1173 group showed the lowest IRE1 expression followed by the shIRE1 2219 and shIRE1 2732 groups (Figure 3(c)). The recombinant pEGFP-N1-ATF6 and pEGFP-N1-PERK plasmids were used to overexpress ATF6 in rMCs (*p* < 0.05, Figures 3(d) and 3(e)) compared with the normal group, and elevated levels of IRE1 mRNA were detected in rMCs transfected with pEGFP-N1-IRE1 (Figure 3(f)).

### 3.4. Regulation of ERS Signal Pathways Modulated ATF4 Expression in rMCs

**Figure 4:**
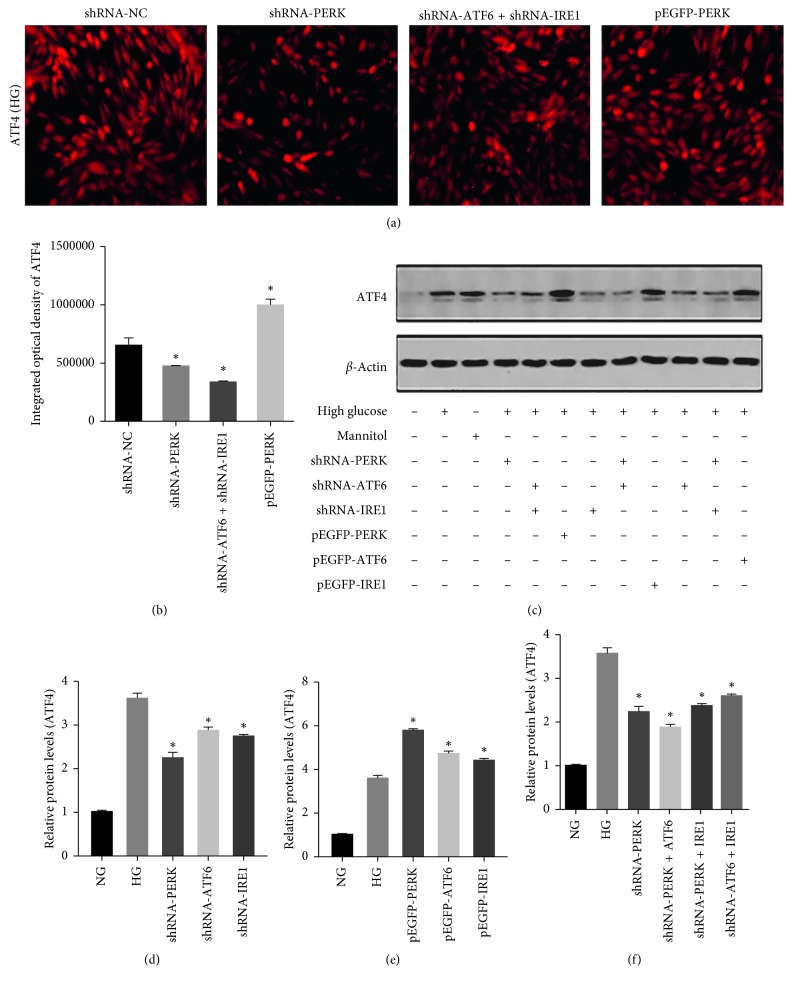
Immunofluorescence and immunoblotting of ATF4 in rMCs at HG. (a, b) Inhibition of UPR pathways significantly decreased ATF4 immunofluorescence. (c–f) The protein levels of ATF4 in rMCs transfected with PERK, ATF6, and IRE1 shRNAs or overexpression plasmids at HG for 48 hours were measured by western blot analysis. The results were representative of 3 independent experiments (*n*=3; ^*∗*^*p* < 0.05).

To examine the effects of the 3 ERS pathways on Müller cells, we treated rMCs with 3 different specific shRNA constructs. Both the immunofluorescence and protein levels of ATF4, a downstream target of PERK, were decreased significantly in HG-exposed in cells transfected with PERK-shRNA (*p* < 0.05, Figure 4). We next investigated whether inhibition the ATF6 and IRE1 pathways downregulated PERK. The results of immunoblotting analysis indicated that knockdown of ATF6 or IRE1 significantly decreased ATF4 expression in Müller cells under HG condition (*p* < 0.05). In order to identify the effects of overexpression of these proteins, rMCs exposed to HG were transfected with pEGFP-ATF6, pEGFP-PERK, and pEGFP-IRE1, respectively. rMCs transfected with pEGFP-PERK showed the highest expression of ATF4 after HG treatment (Figures 4(c) and 4(e)). To gain more insight into the 3 UPR channels, we inhibited 2 of the 3 pathways in different combinations. Inhibition of both ATF6 and IRE1 resulted in a significant decrease of ATF4 compared with PERK-shRNA alone (*p* < 0.05, Figures 4(a) and 4(b)). The results of WB demonstrated that ATF4 protein levels in the shPERK + shATF6 group were significantly decreased compared with shPERK alone. However, the expression of ATF4 in the shPERK + shIRE1 and shATF6 + shIRE1 groups showed no significant difference with the shPERK group (*p* > 0.05, Figures 4(c) and 4(f)).

### 3.5. Regulation of ERS Signal Pathways Modulated XBP1 Expression in rMCs

**Figure 5:**
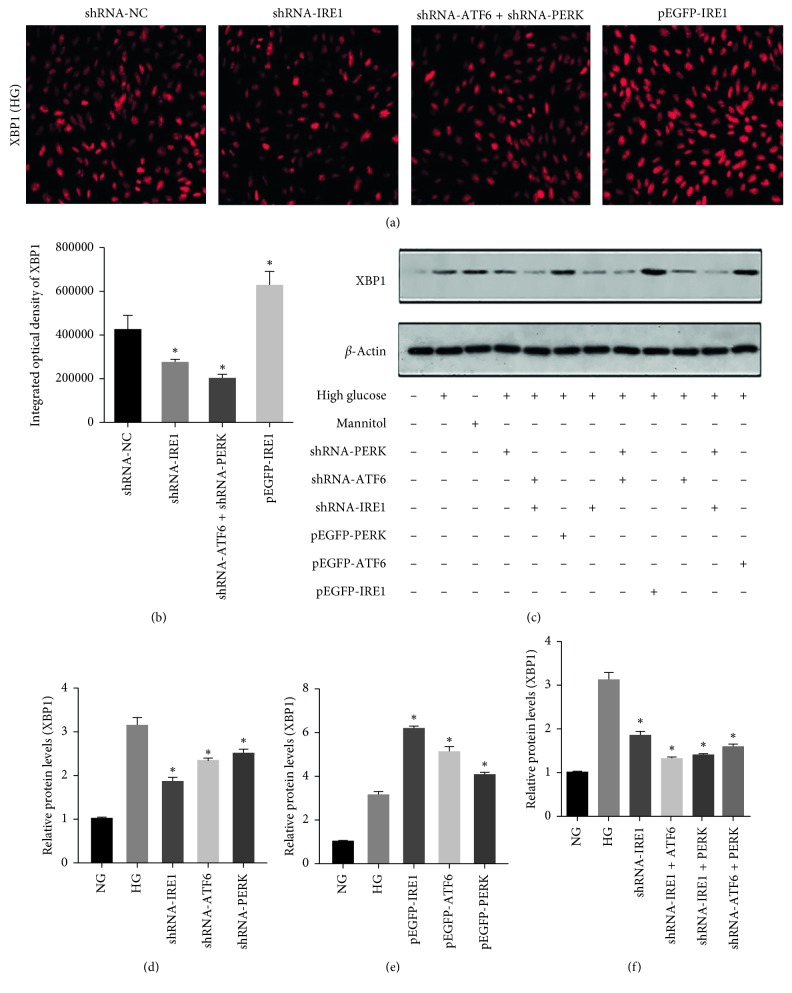
Immunofluorescence and immunoblotting of XBP1 in rMCs at HG. (a, b) Suppression of UPR pathways significantly decreased XBP1 immunofluorescence. (c–f) The protein levels of XBP1 in rMCs transfected with PERK, ATF6, and IRE1 shRNAs or overexpression plasmids at HG for 48 hours were measured by western blot analysis. The results were representative of 3 independent experiments (*n*=3; ^*∗*^*p* < 0.05).

Similar results were observed for XBP1 expression. XBP1 expression in cells with shIRE1 transfection was evaluated by WB and immunofluorescence after exposure to HG for 48 hours. XBP-1 was downregulated (Figures 5(a) and 5(b)). However, rMCs with shRNA transfection exhibited higher levels of these markers compared with normal glucose. Both shPERK and shATF6 inhibited XBP1 (*p* < 0.05), but there was no difference between these groups (Figures 5(c) and 5(d)). rMC transfected with pEGFP-IRE1 showed the highest expression of XBP1 in HG (Figures 5(c) and 5(e)). XBP1 in the shIRE1 group was significantly upregulated compared with the shIRE1 + shPERK and shIRE1 + shATF6 groups (Figures 5(c) and 5(f)).

### 3.6. Regulation of UPR Signal Pathways Modulated ATF6 Expression in rMCs

**Figure 6:**
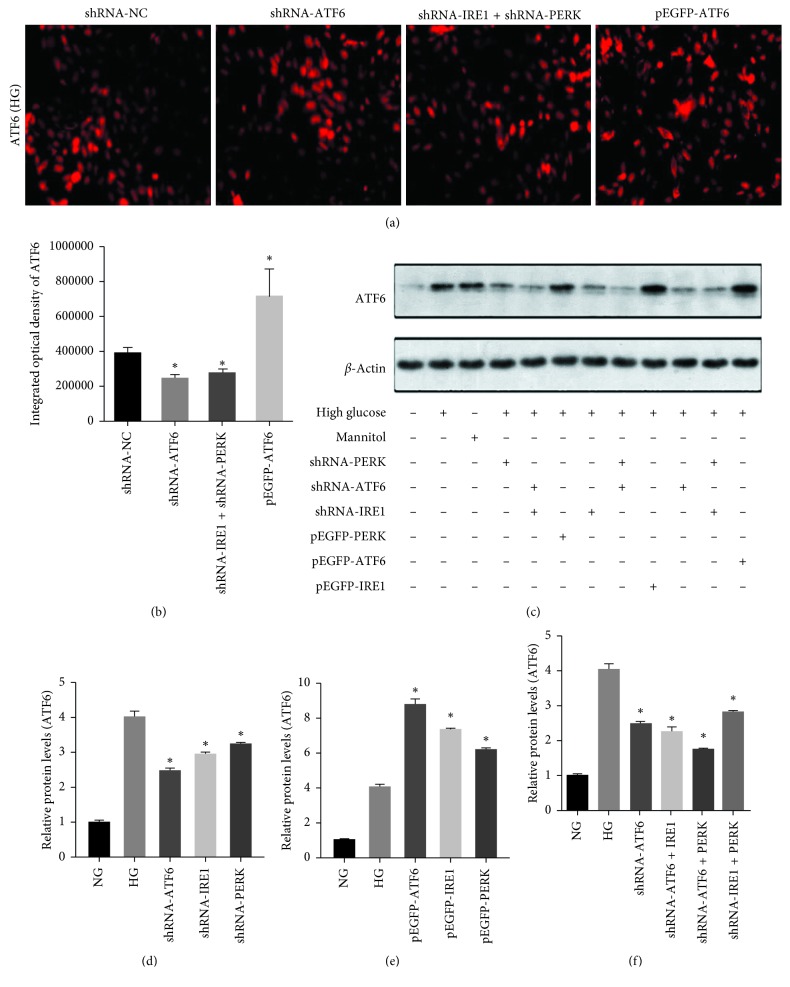
Immunofluorescence and immunoblotting of ATF6 in rMCs at HG. (a, b) Suppression of UPR pathways significantly decreased ATF6 immunofluorescence. (c–f) The protein levels of ATF6 in rMCs transfected with PERK, ATF6, and IRE1 shRNAs or overexpression plasmids at HG for 48 hours were measured by western blot analysis. The results were representative of 3 independent experiments (*n*=3; ^*∗*^*p* < 0.05).

ATF6 shRNA significantly decreased ATF6 levels in rMCs after exposure to HG for 48 hours measured by both immunofluorescence and WB (Figure 6). Similarly, shPERK and shIRE1 remarkably downregulated ATF6. Moreover, ATF6 levels were not significantly different between the shATF6 and shPERK + shIRE1 groups. The expression of ATF6 in the overexpression groups was significantly higher than that in the empty plasmid group (*p* < 0.05, Figures 6(a) and 6(b)). ATF6 was higher in rMCs transfected with pEGFP-ATF6 compared with pEGFP-PERK and pEGFP-IRE1 (*p* < 0.05, Figures 6(c) and 6(e)). When inhibiting any of the 2 signaling pathways, the ATF6 levels in the shATF6, shATF6 + shPERK, and shATF6 + shIRE1 groups were 2.47 ± 0.07, 1.73 ± 0.04, and 2.24 ± 0.14, respectively (Figures 6(c) and 6(f)).

### 3.7. Regulation of ERS Signal Pathways Modulated VEGF and GRP78 Expression in rMCs

**Figure 7:**
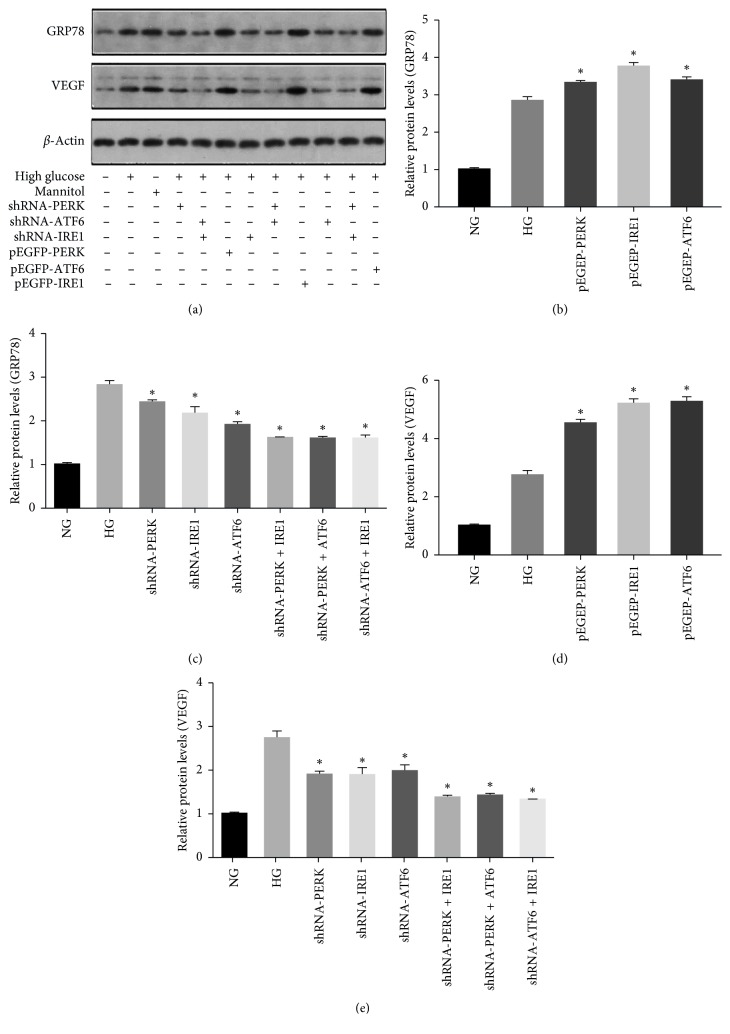
GRP78 and VEGF in rMCs transfected with shRNAs or overexpression plasmids. The expression levels of GRP78 (a, b, c) and VEGF (a, d, e) were measured by western blot analysis. Representative blots from 3 independent experiments (*n*=3; ^*∗*^*p* < 0.05).

Interestingly, the protein levels of GRP78, an upstream target of UPR, were increased significantly in rMCs transfected with the overexpression plasmids after exposure to HG for 48 hours (Figures 7(a) and 7(b)). The expression of GRP78 was significantly reduced. The strongest inhibition of GRP78 was observed in the shATF6 group. When any 2 UPR pathways were suppressed, the expression of GRP78 was lower than that of single inhibition groups. However, the expression of GRP78 among the 2 pathway inhibition groups was not significantly different (*p* > 0.05, Figures 7(a) and 7(c)). Similar results were obtained for the expression of VEGF. Overexpression of any ERS pathway in rMCs induced a statistically significant increase of Müller cell-derived VEGF in response to HG. No significant difference of VEGF levels was observed between the pEGFP-IRE1 and pEGFP-ATF6 groups (*p* > 0.05). pEGFP-PERK induced significantly less increase than the other pathways (Figures 7(a) and 7(d)). The VEGF protein levels were reduced to the same extent (*p* > 0.05) by inhibition of any of the 3 signaling pathways (Figures 7(a) and 7(e)).

## 4. Discussion

Compelling evidence suggests that ERS plays an important role in chronic inflammatory conditions such as diabetes [27]. However, how ERS in rMCs under HG promotes VEGF expression remains poorly understood. Previous studies showed that Müller cells are capable of basal VEGF secretion and of VEGF synthesis in HG [23, 28, 29].

In the presented study, we developed Müller cell lines *in vitro* and set up a hyperglycemia model to simulate the diabetic environment. Many papers showed that ERS signals and inflammatory factors significantly increased in Müller cells at HG for 48 or 72 hours [18, 19]. Our results showed that the protein levels of GRP78, ATF6, XBP1, and ATF4, as well as VEGF, were increased in HG at 48 hours, indicating the presence of ERS. Of note, the rMCs exposed to HG for 48 hours appeared to exhibit higher activation of the ATF6 than PERK and IRE1 pathways (as measured by ATF4 and IRE1, respectively). In addition, overexpressing any pathway caused more increase in ATF6 compared with PERK and IRE1. These data indicated that the ATF6 pathway was more sensitive to HG.

To examine the relevance of glucose in the hypertonic solution, experiments were performed using mannitol (25 mmol/L) as a hypertonic compound with normal glucose. The results demonstrated that the high-mannitol did also induce a rapid upregulation of ERS markers, suggesting that the ERS was merely induced by the hypertonic solution and was only partially due to glucose in Müller cells. It is an interesting finding, and further consideration was required.

To investigate the individual effects of the 3 UPR pathways, we constructed PERK, ATF6, and IRE1 shRNAs and overexpression plasmids for Müller cells. Our results showed that the 3 UPR pathways were activated at the same time when exposed to HG for 48 hours. Previous studies showed that activated PERK inhibited the initiation step of mRNA translation via phosphorylating eukaryotic initiation factor 2*α*. Activated ATF6 translocated to the golgi complex to regulate the expression of molecules involved in protein quality control. In addition, ATF6 promoted IRE1a-mediated splicing of X-box binding protein 1 and its expression [30]. Indeed, we founded that the role of the 3 signaling cascades was not independent after exposure to HG for 48 hours.

In addition, we observed significantly increased levels of GRP78 protein in the overexpression groups, suggesting a positive feedback loop. Furthermore, significant upregulation of PERK, IRE1, and ATF6 pathways might induce accumulation of unfolded or misfolded proteins in the endoplasmic reticulum, leading to the endoplasmic reticulum stress response and increasing GRP78 protein levels. An interesting finding of our study was that UPR branches mediated by IRE-1 had a substantially greater effect on GRP78 expression compared with PERK and ATF6 pathways. Concurrently, GRP78 expression was further decreased when any 2 signaling pathways were suppressed.

Moreover, we demonstrated that regulation of the 3 UPR signaling pathways effectively modulated the production of VEGF. Moreover, our study showed that transfection with PERK, ATF6, and IRE1 shRNAs markedly attenuated VEGF expression in Müller cells compared with the control group, while no significant differences in the levels of VEGF between the 3 shRNA groups. These observations indicated that the sensitivity of the ERS-triggered pathways was quite similar.

## 5. Conclusion

In summary, our data demonstrated that the high glucose or high osmotic pressure produced by HG activated ERS in Müller cells. Regulation of the 3 UPR signal pathways effectively modulated the other 2 signaling cascades and the production of VEGF in Müller cells. Inhibition of these 3 UPR pathways might be a potential therapeutic target for DR.

## Data Availability

The datasets used and/or analyzed during the current study are available from the corresponding authors on reasonable request.

## Abbreviations

ERS: Endoplasmic reticulum stress
DR: Diabetic retinopathy
VEGF: Vascular endothelial growth factor
PERK: PKR-like endoplasmic reticulum kinase
IRE1: Inositol-requiring enzyme 1
ATF6: Activating transcription factor 6
ATF4: Activating transcription factor 4
XBP1: X-box binding protein 1
UPR: Unfolded protein response
rMC: Rat Müller cell
DMEM: Dulbecco's modified Eagle's medium
GFAP: Glial fibrillary acidic protein
HG: High glucose
shRNA: Short hairpin RNA
SDS-PAGE: Sodium dodecyl sulfate polyacrylamide gel electrophoresis
qRT-PCR: Quantitative real-time polymerase chain reaction
SD: Standard deviation
ANOVA: Analysis of variance.
